# Light People: Academician Songlin Zhuang

**DOI:** 10.1038/s41377-022-00775-y

**Published:** 2022-04-21

**Authors:** Hui Wang, Cun Yu, Naifei Guo

**Affiliations:** Changchun Institute of Optics, Fine Mechanics and Physics, Chinese Academy of Sciences, 3888 Dong Nan Hu Road, 130033 Changchun, China

**Keywords:** Optical spectroscopy, Terahertz optics

## Abstract

Can you imagine that one single small capsule can achieve navigation and positioning, and do gastroscopy of the digestive system? Can you imagine that a machine can automatically complete the task of analyzing more than 4800 new coronavirus samples within 24 h? Don’t be surprised, this has become a reality, thanks to Academician Songlin Zhuang and his team, who applied terahertz technology to various fields such as medicine, food safety, ecological environment monitoring, and social security. Dedicating his entire life to optics and terahertz, Academician Zhuang personifies courage, determination and persistence. He believes in science and hard work. He also takes it as his responsibility to pass on the optical spirit and cultivate optical young scientists.

Today, Light Special Correspondents will take you to the University of Shanghai for Science and Technology to see the beginning and experience of Academician Songlin Zhuang’s optical road. I believe it will open a window to a different optical world, and allow our readers to appreciate its unique beauty.

## Abstract (web summary)

As a “pioneer” in the field of terahertz in China, Academician Songlin Zhuang has cultivated many constructive results on this “Frontier”, let us to see his optical road.

## Graphical abstract

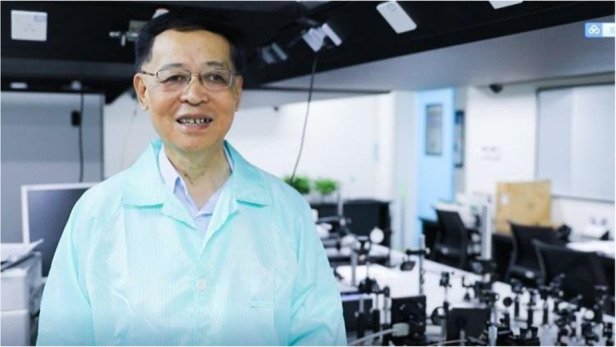

**Figure:**
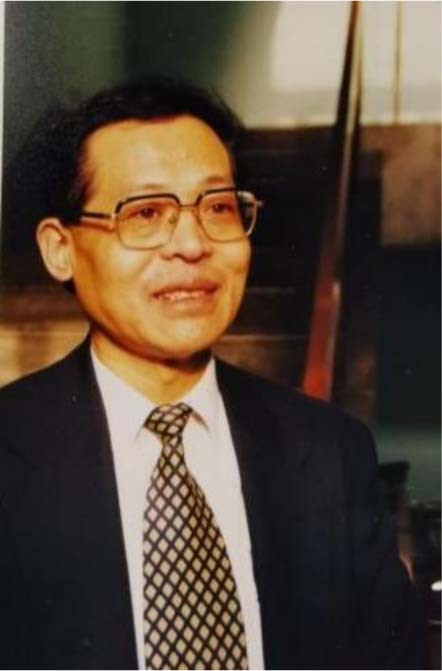

Songlin Zhuang graduated from the Fudan University in 1962. He went to the United States for research in 1979. He obtained the Ph.D. degree from the Department of Electronic Engineering of the Pennsylvania State University in 1982. He was rated as a national-level young and middle-aged expert with outstanding contributions in 1984 and won the first prize of the Shanghai Science and Technology Elite Nomination Award in 1989. In 1995, he was elected as an academician of the Chinese Academy of Engineering. He used to serve as the chairman of the Chinese Instrument and Control Society and the director of the Chinese Optical Society. He has designed more than one hundred optical systems and instruments, and is the first researcher to develop optical system CAD in China. He presided over the completion of the largest optical instrument design software system in China, and has carried out domestic pioneering work in optical imaging psychophysical experimental research and made a comprehensive and systematic research on incoherent optical information processing and rainbow holography technology. He is known as “one of the main contributors of modern white light information processing”. In recent years, the key laboratory led by him has made outstanding contributions to the research and industrialization of international frontier fields such as terahertz technology, optical super-resolution imaging, micro-nano optical engineering and medical optical engineering. He has published more than 300 papers in peer-reviewed journals including *Nature Photonics, Nature Communication, PRL, Opt. Lett., Opt. Exp*, etc., and published one book “Optical Transfer Function”.

**1. For a long time, terahertz has been regarded as the** “**Last Frontier**” **of the electromagnetic wave band due to the lack of effective excitation methods. As the “captain” in the field of terahertz, you have cultivated many constructive results on this** “**Frontier**”**. Can you introduce its current applications and future development trends?**

Terahertz has developed very rapidly in the past 20 years. We know that terahertz is an electromagnetic wave band between far-infrared waves and millimeter waves. Its frequency is lower than infrared while higher than millimeter waves. Most biological macromolecules have their vibrational rotation spectra in the terahertz band, so the fingerprint characteristics of terahertz will be better. But why hasn’t terahertz been developed for a long time? It is mainly affected by three factors. The first one is the lack of a suitable terahertz light source; the second one is the lack of good receivers and detectors; and the third one is the lack of functional devices such as terahertz transmission, focusing and filtering. Since the 1990s, with the development of these three aspects, the complete terahertz system and the market have also developed.

I think the current terahertz development is just like an airplane in the acceleration phase on the runway, and some functions have been practically applied. When the acceleration reaches a certain speed, it can take off. Once it takes off, its industrialization development will be very rapid.

At present, terahertz has been applied in many fields, such as biomedicine, public safety, communication, ultra-precision optical inspection, medical optical equipment, etc. Some applications have already been used in cooperation with hospitals and entered people’s lives. Let me give you a few examples that closely relate to our life.

One example is the application of terahertz in public security. In the past, traditional hand-held metal detection was a common security check method used in the field of public security. With terahertz detectors, fast security inspections with no contact, no stopping, and no radiation can be achieved. Shanghai, Shenzhen and other places have begun to use this kind of detectors. It can also be used in customs inspection of letters containing contraband. In the past, people used the dogs’ sense of smell to detect dangerous items, but now terahertz instruments can be used to find suspicious items. Terahertz has brought revolutionary changes to security.

For another example, the use of terahertz radiation can determine the boundaries of brain tumors, thereby improving the effect of brain tumor treatment. Since glioma is very similar to normal cells, the boundary between the two is very blurred. If the brain tumor is not cut enough, it will cause the patient’s disease to relapse, but if it is cut too much, it will be a kind of damage to the patient’s body. The use of terahertz can detect the marginal zone of glioma, which greatly improves the accuracy and efficiency of surgery. At present, this result has been applied to Huashan Hospital, affiliated to the Fudan University, and the clinical results are very effective.

In addition, terahertz imaging and spectroscopy technology can also be applied to the detection of kidney diseases, such as kidney stones. We know that kidney stones are formed by the accumulation of salt and minerals in the body. One of the main methods to capture and prevent the agglomeration is to detect the metabolites that produce agglomeration in the urine, but this process is time-consuming. Terahertz can be used for accurate detection, prescribing the right medicine, and shortening the detection cycle.

We are also cooperating with the Ruijin Hospital, affiliated to the School of medicine, Shanghai Jiaotong University, hoping to apply terahertz to pre-detection of myocardial infarction. In the past, in order to predict myocardial infarction, it was necessary to collect a large amount of blood and the process was also time-consuming and expensive. So the pre-detection of myocardial infarction could not be widely used. However, terahertz technology can make the pre-detection of myocardial infarction consume less blood, less time, lower cost, and higher efficiency.

In addition to the above applications, terahertz waves provide greater possibilities for space communications and the Internet of Everything, such as the current advanced 5G and future 6G communications. 6G is estimated to fall in the terahertz band. What 6G wants to achieve is not only the increase in network speed, but also the study of the interaction between electromagnetic waves and human consciousness. However, there are many problems to be solved in order to achieve this application. For example, the higher the frequency, the denser the transmission network and the more base stations are needed. Terahertz needs transmitting in the air, but as it is very afraid of water, I think it might be better to use it in adjacent spaces.

**2. At the beginning of the epidemic, you and your team undertook the emergency project of the Chinese Academy of Engineering and made important contributions to fight against the epidemic. Can you briefly describe it?**

At the beginning of the outbreak, we were entrusted with a mission at a critical and difficult moment. At that time, my team and I successfully developed a fast and fully automatic detection system that can detect 4,800 new coronavirus samples within 24 h. This is the first generation. Now the improved second-generation equipment has been successfully developed. This equipment is equivalent to a P2 + laboratory, which can realize fully automatic non-contact detection, reduce the risk of doctors’ infection, and play a very important role in the prevention and control of the epidemic.

**3. You actively promote the transformation of scientific research results. But there is a big difference between scientific research and transformation of results? Can you talk about your experiences and feelings?**

I have a background in physics, so I always want to do basic research work. Although basic research is very important, technology is ultimately to serve the development of society and economics. To truly solve a series of “stuck neck” problems, our team is now working hard to move the development of terahertz from the research stage to applications.

I have always insisted on this point of view: doing scientific research is not to do what we want. It requires us to think clearly about every step before research. For example, why do it? How to do it? How to use it after it is finished? The ultimate goal of doing research is to serve people and society. If just for publishing a few papers, I think it is meaningless. When I invent an instrument for biological detection, making it a testing device for patients is the ultimate goal. In 2009 Guohua Xiao, who had come back from the United States, proposed to study a navigable capsule endoscope that can be used for gastroscopy. I thought it was feasible, so I used my own funds to support it and at the same time helped to find other investments. The largest investor was Tsinghua Tongfang. We know that traditional gastroscopy is uncomfortable, so many people are discouraged. According to the survey, more than half of people with stomach discomfort are afraid of endoscopic examination. And because of this fear, many people missed the best time for treatment. Painless gastroscopy also has a problem, it requires injection of anesthetic. Many elderly people are reluctant to do it because the cognitive level of the brain will be greatly reduced and some even become dementia after anesthesia injection. It has a great impact on the elderly, and young people are also advised to use with caution The “Capsule Gastroscope” developed by us is very simple to use. The patient only needs to take a small capsule to complete the examination. The front end of the capsule endoscope is a miniature digital video camera with multiple flashes, which can take clear pictures in the dark digestive tract. Doctor can view the patient’s digestive tract in real time on the computer screen. In addition, this capsule also has a positioning function. After swallowing the capsule the patient lies on the examination bed, the doctor can control and navigate the capsule through the external magnetic field and guide it to move around in the patient’s body. It has 6 “viewing angles” such as looking up, overlooking, rotating, etc, so the doctors can diagnose patients from multiple dimensions. This achievement has now entered medical insurance, and more than 700 hospitals across the country have done more than 1 million cases and achieved very good results.

**4. A lot of researches you have done are applying terahertz to the field of life and health. Since medical devices require a license, what preparations do you have before entering the clinic?**

Yes, medical devices must have a license. We promptly applied for licenses from all levels of medical license issuing agencies and inspection and testing agencies. They were very supportive of our work. For example, the capsule gastroscope I just mentioned was developed in cooperation with Academician Zhaoshen Li of the Changhai Hospital of Shanghai Second Military Medical University.

Papers are also a focus of our work. In recent years, we also put a lot of effort in writing papers in the world’s leading academic journals. We published a series of articles in *Nature Photonics, Light: Science & Applications, Nature Communication, Science and Advanced Material* et al. Industrialization is always our ultimate goal. For instance, Professor Qiwen Zhan of our team, one of his researches is beam regulation and photon orbital angular momentum being rated by the United States as one of the top 30 optical advancements in the world in 2020. His research results will have important applications in the future. In addition, our team also does some basic research, such as the problem of human energy conversion. Why can we obtain huge energy for the normal functioning of the human body through eating? Why is the efficiency of the chemical reaction in the human body inconsistent with the reaction in the beaker? There are 125 basic scientific questions in Science, 3 of which involve biochemical questions. We are also studying, for example, why people need sleeping? What does sleep adjust for human? How long do people need to sleep, etc. The corresponding research was published in top academic journals such as *PNAS and Nano Research*.

In addition, a lot of our work is also related to the actual needs of the society. The whole world is still under the epidemic. Every day the number of people entering Shanghai is around 3000–4000. These people need nucleic acid test. This is a very difficult task, so the relevant departments contacted us to explore whether the nucleic acid test can be carried out like a security check in public. In response to this problem, we made a bold conjecture that is to develop a resonance chip replicating the virus by breathing on the chip, and then use terahertz for detection. This method is definitely better than the current nasopharyngeal swab. It is faster and more comfortable. This research is still in the laboratory stage at present. We hope that it can be practically applied in the future to benefit all mankind as soon as possible.

**5. Your achievements in the field of optics are obvious, but few people know that you, as a programming** “**layman**”**, designed the first optical system optimization program in China, making the amount of calculation that would have taken a whole year to complete, now can be obtained in one day. Can you talk about the difficulties encountered when doing this research and how to overcome them?**

In 1962, after graduating from the Fudan University, I was assigned to the Shanghai Optical Instrument Research Institute to do optical design work. In fact, optical design is to design dozens of parameters such as radius, refractive index, and air gap to obtain an optical system with excellent imaging quality. At that time, it took 6 min to calculate a light path with a hand-cranked computer, and two people were needed to verify the calculation. So the design of a complex lens took one year. I thought this was an optimization problem with boundary conditions. In 1962, the American Journal of *Applied Optics* was founded. The first issue was to introduce automatic optical design. I felt that this coincided with my idea, so I tried to do it by calculating first and then optimizing. At that time, the computer was not as developed as it is now. It could only calculate 70 operations per minute, and it took about 7 s to perform a division operation. It took me half a year to finally design the optical system program, which was verified to be accurate. This is the first program I wrote. Later, I cooperated with Prof. Zhicheng Weng from the Changchun Institute of Optics, Fine Mechanics and Physics (CIOMP) to optimize the program, making this program widely used. I have a close relationship with CIOMP. The first person I cooperated with was Prof. Zhuying Jiang. Later I also served as a member of the Academic Committee of the State Key Laboratory of Applied Optics. The director at that time was Mr. Guoguang Mu.

**Figure:**
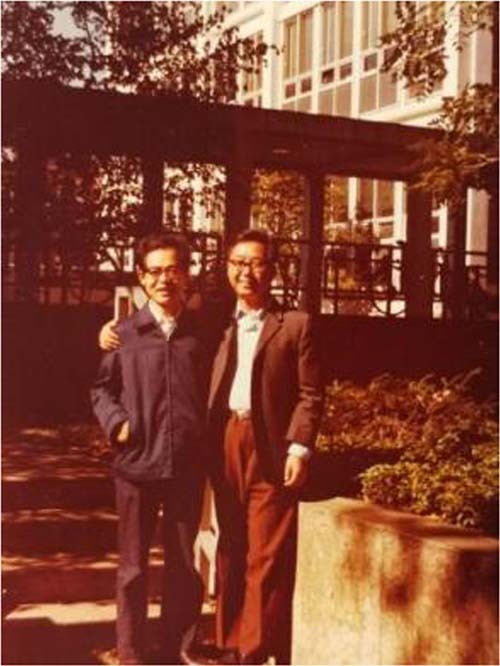
Songlin Zhuang and Guoguang Mu in the Department of Electronic Engineering, University of Pennsylvania in 1980

**6. You once said that your favorite major is actually theoretical physics. But by coincidence, you were assigned to the optics major. How did you adjust your mentality? What do you think of optics now?**

When in high school, I was good at math and won the Shanghai Mathematics Competition. Originally I wanted to study at the Peking University. But my parents did not want me to go to another city, so I gave up. In the second year of high school, Zhenning Yang and Zhengdao Li won the Nobel Prize in Physics. This incident touched me a lot. Therefore, I applied for the Fudan University when I was in my third year of high school. Studying theoretical physics is my biggest wish. But in the division of majors, I was assigned to the optics. At that time, compared to radio physics, nuclear physics and solid-state physics, optics belonged to a relatively “old” discipline. So I ran to ask the dean of the department, he said that the optics major is the strongest of all the physics majors in the university. In the end, I accepted this reality and settled down.

There was also an episode when I was in college. I took a semester off due to illness so I should repeat a year. I asked the dean of the department if there was the possibility that I would not repeat. He said that I don’t need to repeat if I could pass the exam. So I taught myself Advanced Optics and other courses at home. Later I got 5 points in all courses, and finally I did not repeat and graduated successfully. After graduation, I applied for the postgraduate program of Mr. Daheng Wang. However, due to physical reasons, I stayed in Shanghai and met Biwen Zhu, the director of the Shanghai Institute of Optical Instruments. She said that the Shanghai Institute of Optical Instruments also did applied and fundamental research, such as optical information processing, although it belonged to the Ministry of Industrial and focused on the product development and technical research. So I decided to stay here and this period helped me a lot in my understanding of optical instruments.

**Figure:**
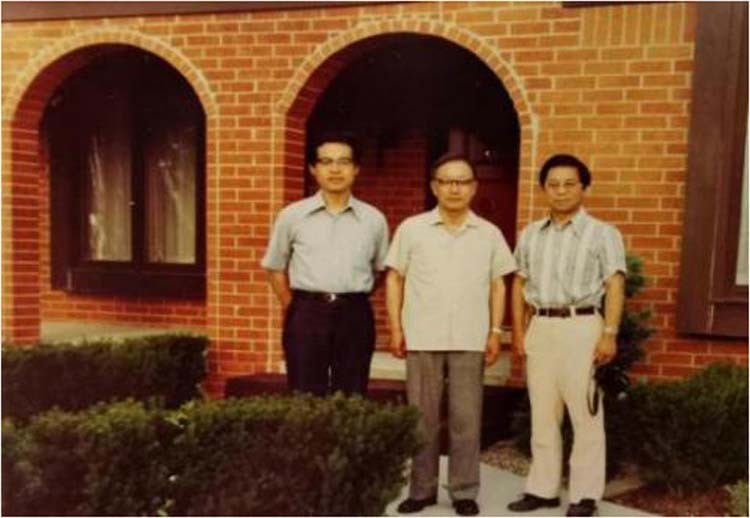
Songlin Zhuang with Daheng Wang and Zhengding Wang in Detroit in 1980

**Figure:**
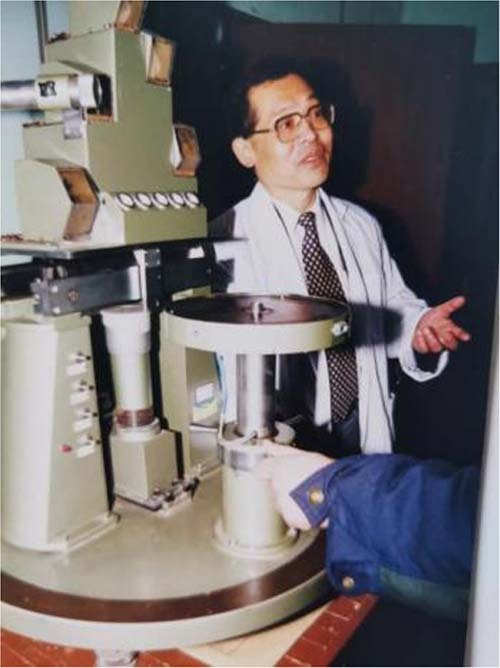
Songlin Zhuang at the Shanghai Institute of Optical Instruments

**7. You started as a student, became the director of the Shanghai Institute of Optical Instruments, and later became an academician of the Chinese Academy of Engineering and the dean of the School of Optoelectronic Information and Computer Engineering, University of Shanghai for Science and Technology. How has your perception of scientific research changed over the years?**

First of all, I am a scientific and technological researcher, and my idea is that I should always stand on the front line of scientific research. You can do what others have done, but you need to dig some new content, do things that have new breakthroughs and innovations. When I gave a lecture to the middle school students, I said that I hope they could cultivate their own innovative spirit. For example, when you solve plane geometry problems, you have to come up with some ingenious methods. The method I used to solve problems in middle schools often different from others. Another point I want to share with the young researchers is that when we read literature, we should not be constrained by literature. First don’t look at how others do it, but look at what the purpose is, and think about how to operate it yourself, so that you can know where your problems are. Now this era is dominated by young people, and our group of old scientists are actors who are about to call their curtains. What we can do is being willing to be the heroes behind the scenes, creating conditions and providing platforms for young people, leaving the center of the stage to them, and letting them display their talents.

**Figure:**
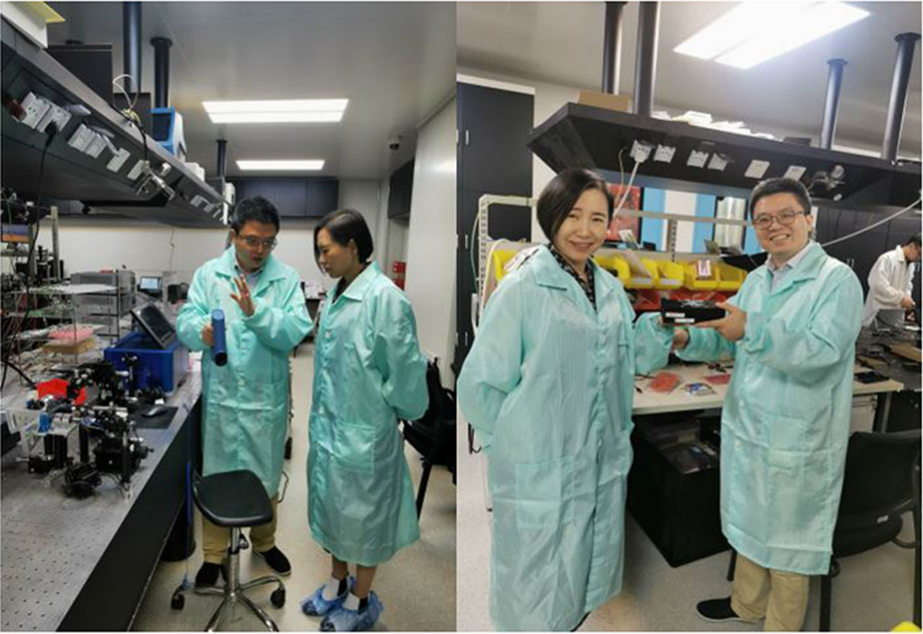
Professor Yiming Zhu introduced the laboratory to Ms. Yuhong Bai, the Director of the Light Publishing Group

**8. People say, “It’s good to enjoy the shade under the big tree”. Many young people may have more opportunities under the “big tree**” **than others, but what advice do you have for those who have not walked into the** “**big tree**”**?**

It is undeniable that this statement has some validity. Following first-class scientists, young people can indeed get more resources. But all things depend on the combination of all the factors like time, place and people. One factor alone is not enough. I think personal effort is the most important thing.

Like our terahertz laboratory, which is very large and world-class, has a lot of funding. Many outstanding scholars decided to work here and we provided excellent experimental environment for them. Moreover, in the application of talent programs and other funding, they enjoy certain advantages, and we also match the corresponding financial support. But this does not mean that people who came to us will definitely have good results. Take myself as an example, after graduation I did not go to the academy or college. Instead, I went to the industry sector first, but I did not give up. I have always believed that gold will always shine. I enriched myself with reading, and I armed myself with knowledge. So I think in order to succeed, you must be down to the earth and keep working hard. One day you will find that God will not disappoint anyone who persists.

**Figure:**
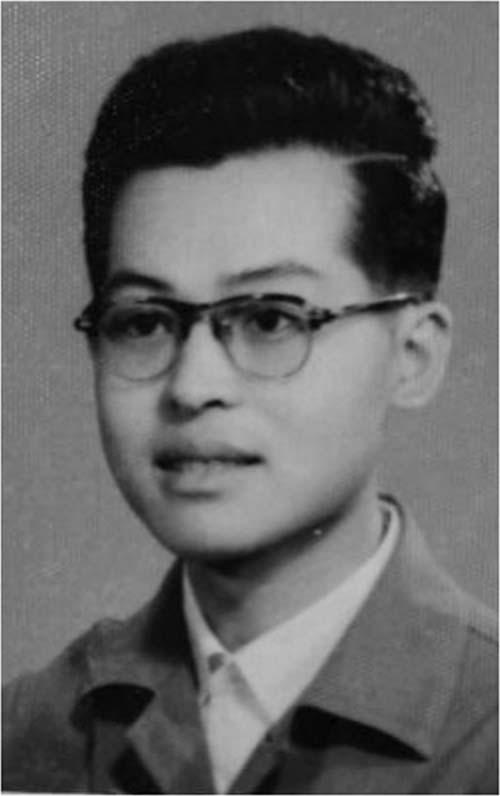
Songlin Zhuang in his youth

**9. Although you are now in your eighties, you are still on the front lines of optics research and teaching. What makes you always devote yourself to it, never admit defeat and persevere in the field of optics research?**

I have an inertial love for optical research. Our laboratory is like my child. Now the area of the laboratory is close to 20,000 square meters. From the beginning of construction, to expansion, and now to growing maturity, the laboratory conditions are getting better and better. Most of the staffs were interviewed by me. Our team is very large, some are from abroad, and some are domestic. The team members are very hard-working and have been recognized by the academic circles internationally and domestically. I feel that it is my responsibility to build this platform well where everyone can show their talents and contribute to the country and society.

**10. In 1982, you resolutely chose to return to China to serve the motherland after obtaining a doctorate in electrical engineering from Pennsylvania State University. After so many years, how do you feel when you look back on the past? Have you thought about staying in the US?**

At that time, the state sent a large number of students to study abroad, and I was one of them. At the age of 38, I was sent by the state to do my Ph.D. at the Penn State University. During my study, my advisors valued me very much and wanted me to stay there after graduation. In fact I was also his best assistant in the lab. During my three-year Ph.D. study, I published more than 30 papers in mainstream international academic journals. However I didn’t think too much about it at the time. I felt that I was sent by the motherland, so how could I not come back? The country sent me out in the hope that I would be successful in my studies and return to the country for its construction. So the day after I received my Ph.D. degree, I called the embassy to help me book a flight back to China. At that time I thought I would never go to America again in my life.

**Figure:**
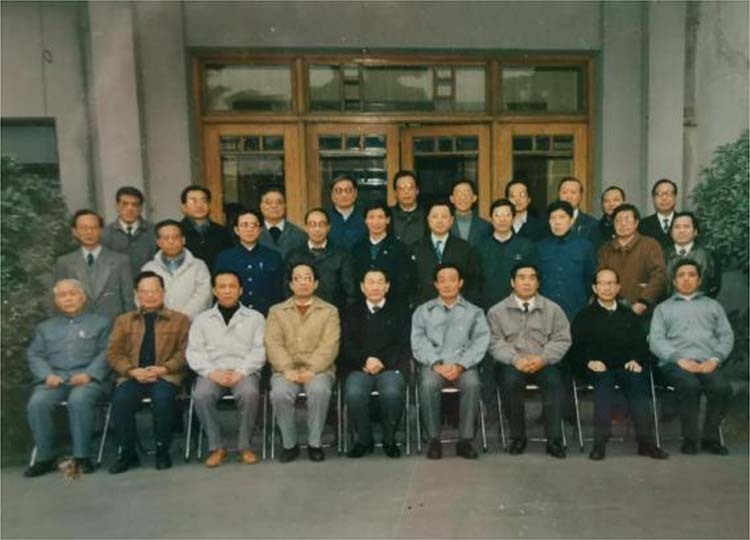
Shanghai leaders and scientists in 1988

**11. You have said that if you have some achievements, half of them should belong to your wife. So what role does family play in your research career and life? How do you balance work and life?**

My wife and I have a pure and deep love starting from childhood. My wife’s parents and my parents are life-long friends. My wife was engaged to me before she was born. But no one cared about this idea later and still emphasized free love. When my mother got sick, my wife (Yiyu Shi) was working in Ruijin Hospital affiliated to the School of Medicine of the Shanghai Jiaotong University. She took great care of my mother. So naturally we fell in love and got married. My wife helped me a lot, not only with her support and understanding for my work, but also with meticulous consideration and concern for my health. From 1979 to 1982, when I visited the United States, the university also set up an office for my wife and invited her to work there. But she could not leave because of the hospital work, so she stayed in China with our two young children. My success is inseparable from my wife’s support and understanding, and I am very grateful to her.

**Figure:**
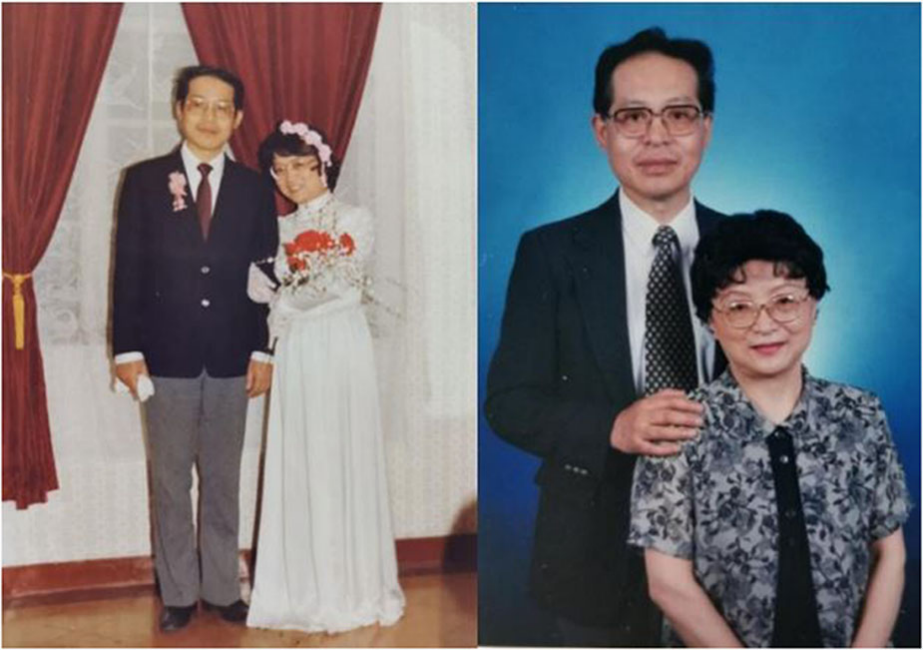
Songlin Zhuang and his wife Yiyu Shi

**12. You love football and have been obsessed with classical music and reading since you were a child. In your opinion, science and art are interlinked. How do you view the relationship between science and art? How do they affect you?**

My hobbies are quite broad. I play the violin since I was a child. When I was in college, I was a member of the school football team, and my wife was a fan. I was also the ambassador of the Shanghai Shenhua football team. In addition, I like classical music very much, like Beethoven’s third, fifth, sixth and ninth symphonies, as well as the works of Borodin in Russia, Bach in Germany, etc. In my opinion, classical music has a special melody beauty, while art and scientific research are sometimes connected as scientific research and exploration can be done better with the infiltration of humanities and arts.

**Figure:**
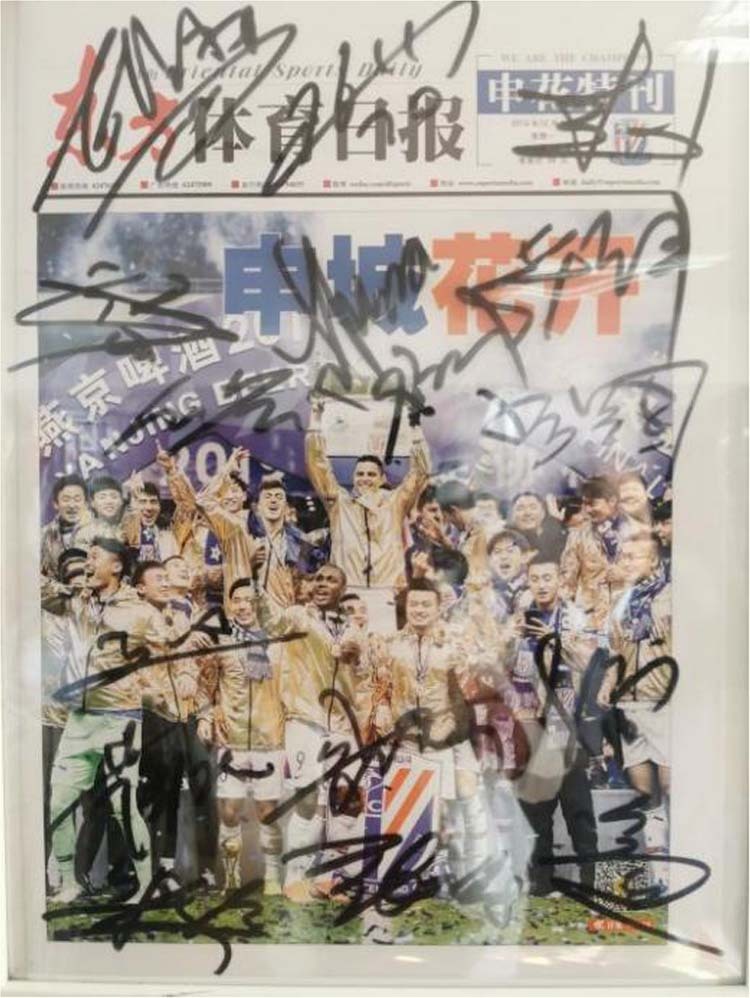
A special issue with team members’ signatures presented to Academician Zhuang by Shenhua Team

**13. Talking about scientific papers, you published your first article “Mode splitting transmission effect of surface wave excitation through a metal hole array”**^1^ **in** ***Light: science & applications*** **(LIGHT) in March 2013. This investigation “may open a door to distinguish the spoof surface plasmons and the coupled modes of surface waves and hole modes.” Two more research papers were subsequently published in 2017**^2^ **and 2019**^3^, **respectively. We all know that LIGHT was founded in 2012. Why were you willing to publish your research results on LIGHT in 2013, which was not well-known at the time? What keeps you publishing papers on LIGHT? Can you talk about your comments and suggestions on LIGHT?**

**LIGHT** is not only well-known in the field of optics in China, but also a famous optical journal in the world. I think like ***Nature Photonics, Light: Science & Applications***, etc. are the world’s top optical journals, so it is my honor to submit and publish the manuscript to **LIGHT**. I remember when I was invited to submit my first manuscript to **LIGHT**, it was rejected. At that time, **LIGHT** just started its publication and I didn’t understand the positioning of this journal. Later when we had new results, we finally published on **LIGHT**. I was very impressed by its strict review standard at the time. I also believe that **LIGHT** will get better and better, as it is now.

**Figure:**
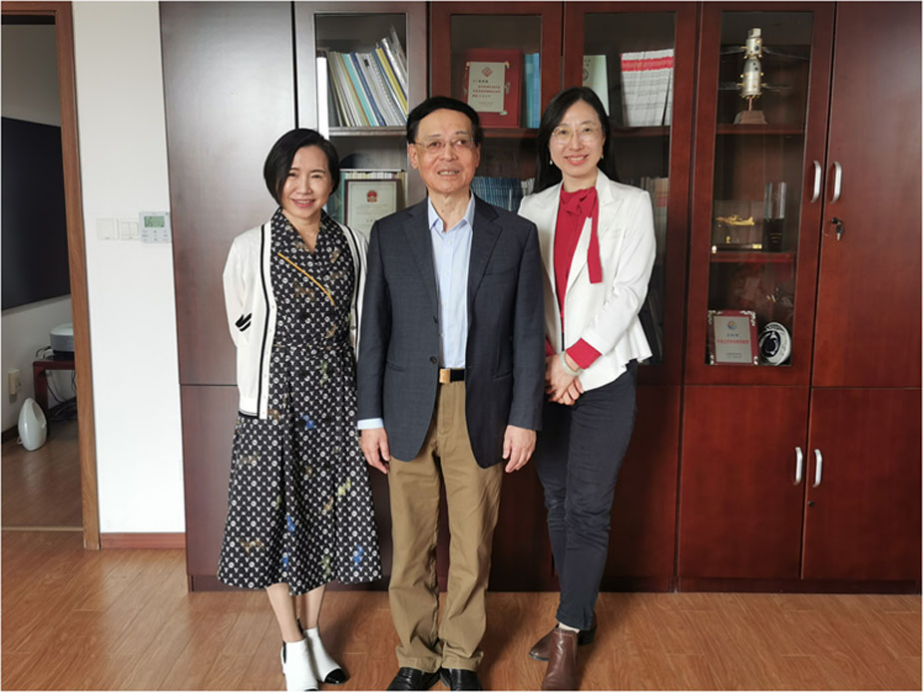
Songlin Zhuang with Ms. Yuhong Bai and Light’s special reporter Hui Wang

**14. In recent years, you have begun to actively participate in popularization of optical science, enlightening the majority of primary and secondary school students. In addition, you give professional introductory course for freshmen, why do you want to do this? What was the original intention?**

I am currently working in the University of Shanghai for Science and Technology, so I often go to the university headquarters and the affiliated middle school to give lectures. Nowadays, young people are faced with many temptations. I hope that through my experience sharing, they will have a right direction. Teaching them how to study, how to identify career development goals, how to establish a correct outlook on the world and life, and cultivating their self-directed learning ability and good study habits are my goals. Sometimes I also do some optics science for them, hoping they like it and finally choose optics as their career.

**15. In your career, who has the greatest influence on you? why?**

On the way of my growth, different people have different influences on me at different stages. For example, in middle school, my teacher had a great influence on me. I clearly remember that most of the teachers who taught us at that time studied abroad before. They were knowledgeable and focused on cultivating students’ learning thinking. At that time, there was a magazine in China called “*Mathematics Newsletter*”, and every issue would give some difficult questions. The name of the person who answered the question would be published in the next issue, so we specially set up an interest group to study and conquered problems together. Later, when I went to the United States, my mentor was Zhenhuan Yang who had a great influence on my academic path. Now every year when he returns to China, I will invite him to Shanghai. For me, he is both a teacher and a friend.

**16. What advice do you have for young researchers?**

The most beautiful scientific research oath is to love the country and the party. I hope that every scientific research worker can adhere to the original intention of serving the country with science and technology, consciously be a model in promoting the spirit of patriotism and hard work, and write a wonderful life in the torrent of the times. I hope that no matter what school or company the scientific researchers are in, they can work positively, make outstanding contributions, and spend more time and energy on scientific research when they are young. Finally, I would like to send you a message of blessing, which is also my code of conduct: it is definitely worthwhile to have a mind higher than the sky, be down to the earth, and work hard persistently.

## Author’s Note

In October 2021, Ms. Yuhong Bai, Director of Light Publishing Group, and I went to the Shanghai University of Science and Technology to interview Academician Songlin Zhuang. “Warm as jade” was my first impression of Academician Zhuang. He treats people with kindness and generosity, which instantly made me felt at ease and less nervous. “a Renaissance Man” was the impression I was left with after communicating with Academician Zhuang. He is by no means an “old pedant” who only concentrates on scientific research, but a well-groomed, stylish gentleman dressed in tailored suits, proficient in violin-playing, good at calligraphy, and likes to play football, a successful man with a colorful life. He and his wife both come from famous families, and are also both well celebrated figures in their respective fields. Talking about his achievements in the field of terahertz, Academician Zhuang told me about his youthful high ambitions, the fruitful achievements he made at middle age, and the students he has taught who are working all over the world today. Listening to Academician Zhuang, I quickly realized that his success is the result of consistent, hard work. In him we see the perfect combination of science and art, virtue and talent. He is not only a pioneer in opening up the field of terahertz, but also a developer of transmitting optical science. He dedicated his life to the research of terahertz, and to the science of optics. But more impressive is his personality. He is not arrogant or impetuous in good times while not discouraged or depressed in adversity, and always follows his heart. He is a perfect role model to young science workers. I hope that one day, when we have reached the age that Academician Zhuang is now, we can also proudly look back at our life’s experiences, raise a glass to ourselves, and say with confidence that we have not wasted our youth, and have lived a full and meaningful life.

**Light special correspondents**

*Hui Wang is the Deputy Director of the Office of International Cooperation in the Changchun Institute of Optics, Fine Mechanics and Physics (CIOMP), Chinese Academy of Sciences (CAS). She currently works on international communication and cooperation for the CIOMP and was a founding member for the Nature Publishing Group and CIOMP joint journal* ***Light: Science & Applications****. She is the founder of* “*Rose in Science*” *and has published several articles in* ***Acta Editologica, International Talent, Light: Science & Applications****, etc., and was invited to take an interview by SPIE Women in Optics, which was published in 2015.*

*Cun Yu works at the Office of International Cooperation in the Changchun Institute of Optics, Fine Mechanics and Physics (CIOMP), Chinese Academy of Sciences (CAS). Her main duties cover the Sino-Belarus International Innovation Center, internation al cooperation projects and exchanges between CIOMP and institutions in Belarus, Ukraine and Russia. She has published multiple articles in the journal International Talent, Light: Science & Applications, and is a member of CSA’s Science & Technology Translations Association.*

*Naifei Guo works at the Office of International Cooperation in the Changchun Institute of Optics, Fine Mechanics and Physics (CIOMP), Chinese Academy of Sciences (CAS). She has studied in Europe for seven years. Now she is mainly responsible for the management of the English website of CIOMP, foreign affairs management, etc.*
